# Unveiling the Drivers of Polio Vaccine Uptake: Insights from a Multi-Country Study of 37 Nations in Sub-Saharan Africa

**DOI:** 10.1371/journal.pone.0316884

**Published:** 2025-03-19

**Authors:** Getayeneh Antehunegn Tesema, Michael Sarfo, Sylvester R. Okeke, Edward Kwabena Ameyaw, Sanni Yaya

**Affiliations:** 1 Department of Epidemiology and Biostatistics, College of Medicine and Health Sciences, University of Gondar, Gondar, Ethiopia,; 2 School of Human and Health Sciences, University of Huddersfield, United Kingdom,; 3 Centre for Social Research in Health, UNSW Sydney, Australia,; 4 Institute of Policy Studies and School of Graduate Studies, Lingnan University, Tuen Mun, Hong Kong,; 5 L & E Research Consult Ltd, Upper West Region, Ghana,; 6 The George Institute for Global Health, Imperial College London, London, United Kingdom; Gabriele d'Annunzio University of Chieti and Pescara: Universita degli Studi Gabriele d'Annunzio Chieti Pescara, ITALY

## Abstract

**Background:**

Childhood vaccination is a highly cost-effective strategy for preventing vaccine-preventable diseases, including poliomyelitis. Despite advancements in vaccination coverage across Africa, polio remains a public health concern. Limited multi-country analyses on oral polio vaccine (OPV) dropout in African nations hinder the development of context-specific interventions. This study investigates OPV uptake and associated factors in sub-Saharan Africa (SSA).

**Methods:**

This study analyzed data from the Demographic and Health Surveys of 37 sub-Saharan African countries, encompassing 60,846 children aged 12–23 months. Multilevel multinomial logistic regression models were employed to explore associations between individual- and community-level factors and vaccination status, categorized as non-vaccinated, dropout, or fully vaccinated. Four nested models were assessed, with the model exhibiting the lowest deviance (-2 Log-likelihood Ratio (-2LLR)) identified as the best fit. Variables with p-values <  0.2 in bivariable analysis were included in the multivariable analysis. The adjusted Relative Risk Ratios (aRRR) with 95% Confidence Intervals (CI) were reported to determine statistical significance and the strength of associations.

**Results:**

Among children aged 12–23 months, OPV1, OPV2, and OPV3 coverage rates were 86.59%, 81.27%, and 68.41%, respectively. The prevalence of OPV dropout and full vaccination in SSA were 19.38% (95% CI: 19.06%, 19.69%) and 67.77% (95% CI: 67.40%, 68.14%), respectively, with a dropout rate of 20.98%. Key factors significantly associated with non-vaccination included maternal education (primary: aRRR =  0.58; secondary: aRRR =  0.64; higher: aRRR =  0.75), household wealth (poorer: aRRR =  0.91; middle: aRRR =  0.82; richer: aRRR =  0.70), maternal age (20–29: aRRR =  0.67; 30–39: aRRR =  0.60; 40–49: aRRR =  0.59), health facility delivery (aRRR =  0.28), media exposure (aRRR =  0.64), marital status (currently married: aRRR =  0.87), parity (2–3 births: aRRR =  1.11), and rural residence (aRRR =  0.73). Regional disparities revealed higher risks of non-vaccination and dropout in Southern, Central, and West Africa compared to East Africa.

**Conclusion:**

This study highlights the multifaceted determinants of oral polio vaccination dropout in SSA. Targeted interventions, such as improving maternal education, enhancing access to healthcare facilities, addressing socioeconomic inequalities, and mitigating regional disparities, are essential to boosting vaccination coverage and preventing polio resurgence. Focused efforts in Western and Central Africa are critical to sustaining and expanding vaccination programs.

## Background

Childhood vaccination remains one of the most cost-effective and essential public health interventions for preventing vaccine-preventable diseases, including poliomyelitis, a formidable viral illness [1]. A 2014 study conducted by the Centers for Disease Control and Prevention (CDC) demonstrated that routine childhood immunization programs in the United States significantly reduced healthcare costs, preventing over 21 million hospitalizations and 732,000 deaths over two decades [2]. Similarly, a study in low- and middle-income countries, including sub-Saharan Africa, emphasized the cost-effectiveness of childhood immunization, projecting returns of $16 in health system savings and $44 in broader economic and social benefits for every $1 invested in vaccines [1].

Oral polio vaccines (OPVs) have been central to global polio eradication efforts due to their ability to induce strong intestinal immunity, thereby inhibiting viral transmission [3]. However, OPVs carry a small risk of vaccine-derived poliovirus (VDPV) in under-vaccinated populations, raising concerns regarding their continued use [4]. In contrast, inactivated polio vaccines (IPVs) pose no risk of VDPV but are less effective at inducing mucosal immunity, more expensive, and logistically challenging to administer [4]. Despite these differences, OPVs remain the preferred option in low- and middle-income countries, given their cost-effectiveness and ease of distribution in resource-constrained settings [5].

Although significant progress has been made in improving childhood vaccination coverage in Africa, polio remains a major public health concern. The World Health Organization (WHO) reported in 2018 that many African countries continue to struggle with meeting vaccination coverage targets. Poliomyelitis, a paralytic viral infection that causes acute flaccid paralysis, remains a pressing issue [6]. While global vaccination efforts have nearly eradicated polio, with Afghanistan and Pakistan being the last endemic countries, a resurgence of the virus has recently been documented in high-income countries such as the United States and the United Kingdom [7–10].

The WHO’s 2020 report highlights the critical role of reducing infant mortality as part of Sustainable Development Goal 3 (SDG 3), which aims to lower infant mortality rates to 25 or fewer per 1,000 live births across African nations [11,12]. However, WHO data from 2022 reveal that one in 200 polio infections results in irreversible paralysis, with 5–10% of those paralysed succumbing to respiratory muscle paralysis. The presence of even a single infected child poses the risk of a global resurgence of polio, with low- and middle-income countries remaining most vulnerable [13].

Recent years have also witnessed growing vaccine hesitancy and controversies across Africa, resulting in delays or refusals of recommended vaccines despite their availability [14]. The COVID-19 pandemic further disrupted routine childhood immunizations, exacerbating vaccine-preventable disease risks. A study by Fahriani et al. reported that approximately 42% of respondents observed temporary closures of local health facilities during the pandemic, with 13.3% indicating their children missed vaccinations [15]. Historical events, such as the 2003–2004 polio vaccine boycott in Nigeria, fueled by rumors and mistrust, underscore the devastating impact of vaccine hesitancy [16]. Moreover, reports suggest rising vaccine decline trends in Africa, posing significant threats to public health [17].

Numerous factors contribute to the decline in polio vaccine uptake. Studies identify fear and the spread of misinformation, including concerns about sterility and perceived government ulterior motives, as significant barriers to vaccine acceptance [18–20]. Bedford et al. highlighted that post-Ebola experiences in Africa negatively influenced polio vaccine uptake [21].

Socioeconomic and demographic factors also shape child immunization patterns, reflecting persistent health disparities. These factors include age, gender, rural residence, maternal and paternal education levels, unemployment, and limited prenatal care access [22–36]. Misconceptions about vaccine benefits, fears of side effects, and traditional or non-religious affiliations have also been linked to lower vaccination rates [22,37].

Despite the growing body of research on factors influencing vaccine uptake, there remains a lack of comprehensive, multi-country analyses focusing on polio vaccine dropouts in African nations. This study seeks to address this gap by investigating polio vaccine uptake and its associated factors in sub-Saharan Africa. The findings aim to inform the development of tailored, evidence-based interventions to mitigate challenges in achieving universal vaccination coverage and sustaining progress toward polio eradication.

## Methods

### Data source and sampling procedure

This study was based on the latest Demographic and Health Survey (DHS) data of 37 sub-Saharan African countries. DHS is a nationally representative survey that collects data on basic health indicators like mortality, morbidity, family planning service utilization, fertility, and maternal and child health-related indicators. The study employed a two-stage stratified sampling technique to select participants. In the first stage, Enumeration Areas (EAs) were randomly chosen. In the second stage, households were randomly selected within these EAs. This two-stage approach began with selecting clusters of households based on probability proportional to estimated population size, followed by a random selection of households within these clusters. Each country’s survey consists of different datasets including men, women, children, birth, and household datasets. We extracted the dependent and independent variables from the DHS data of 37 sub-Saharan Africa (SSA) countries. We obtained these data after registering and submitting the proposal to the measure DHS program. The detailed methodologies are available from the http://www.DHSprogram.com website. For this study, we used the Kids Record dataset (KR file). The KR dataset was obtained after permission was granted from the measure DHS program. These datasets of 37 sub-Saharan African countries were appended together. The details of this survey including survey design, questionnaires, and sampling techniques have been reported elsewhere [38]. A weighted sample of 60,846 children aged 12-23 months in SSA were included in this study (Table 1).

**Table 1:** Number of children aged 12-23 months across included countries in sub-Saharan Africa.

Country	Weighted sample	Percentage (%)
Angola	2,590	4.26
Burkina Faso	1,388	2.28
Benin	2,511	4.13
Burundi	2,681	4.41
DR Congo	1,690	2.78
Congo	906	1.49
Cote d’Ivoire	741	1.22
Cameroon	1,894	3.11
Ethiopia	1,997	3.28
Gabon	585	0.96
Ghana	583	0.96
Gambia	1,455	2.39
Guinea	1,380	2.27
Kenya	3,674	6.04
Comoros	635	1.04
Liberia	937	1.54
Lesotho	313	0.51
Madagascar	2,327	3.82
Mali	2,029	3.33
Mauritania	2,102	3.45
Malawi	3,228	5.30
Mozambique	1,116	1.83
Nigeria	6,142	10.09
Niger	1,030	1.69
Namibia	386	0.63
Rwanda	1,633	2.68
Sierra Leone	1,834	3.01
Senegal	848	1.39
Sao Tome	336	0.55
Swaziland	485	0.80
Chad	1,916	3.15
Togo	708	1.16
Tanzania	2,134	3.51
Uganda	2,858	4.70
South Africa	669	1.10
Zambia	1,890	3.11
Zimbabwe	1,213	2.00
**Total**	**60,846**	**100**

### Measurement of variables

The dependent variable for this study was oral polio vaccination status. Children aged 12-23 months were considered for this study as children by this age had the opportunity to receive the recommended doses of oral polio vaccines. The childhood vaccination schedule recommends a child receive polio 0 at birth, polio 1 at 6 weeks, polio 2 at 10 weeks and polio 3 at 14 weeks. According to the WHO, non-vaccinated for polio is defined as a child aged 12-23 months who has not received any of the four routine doses of oral polio vaccine, dropout if a child has received any dose of oral polio vaccine but did not take all doses [39,40]. The response for oral polio vaccination status was categorized as; non-vaccinated, dropout (partially vaccinated), and fully vaccinated.

Regarding the independent variables, both individual-level and community-level variables were considered.

### Individual-level variables

Maternal age (grouped as 15 – 19, 20 – 29, 30 – 39 and 40 – 49 years), marital status (grouped as never married, currently married and divorced/widowed/separated), household wealth status (grouped as poorest, poorer, middle, richer and richest), maternal educational status (grouped as no formal, primary, secondary and higher education), birth order (grouped as 1, 2-3, 4-5, and ≥ 6), parity (grouped as one, 2-3, and ≥  4), sex of the child (grouped as male and female), perceived distance to the health facility (grouped as not a big problem and big problem), Antenatal Care (ANC) (grouped as no visit, 1-3 and ≥  4), place of delivery (grouped as home and health facility delivery), occupation (grouped as working and not working) and husband education (no, primary, secondary and higher education) were the individual level variables.

### Community-level variables

Residence (grouped as urban and rural) and sub-Saharan African region (grouped as eastern, southern, western and central Africa) were considered as community-level variables.

### Data management and analysis

Data management and analysis were performed using R version 4.3 and STATA version 17 statistical software. All analyses incorporated sampling weights to account for the survey design and non-response bias. Results were summarized and presented using tables, summary measures, and graphical visualizations. The outcome variable, oral polio vaccination status, was categorized into three groups: never vaccinated, dropout, and fully vaccinated.

Given the scale of the outcome variable and the hierarchical nature of DHS data, a multilevel multinomial logistic regression model was fitted to examine the association between individual and community-level variables with non-vaccinated and dropouts using fully vaccinated groups as a reference category. Using multilevel multinomial logistic regression modelling for this study rather than the classical single-level multinomial logistic regression analysis. Firstly, due to the hierarchical nature of the DHS survey, a multilevel multinomial regression model should be used to obtain more reliable estimates of the model parameters and to parameter over-estimation. We used clusters/EAs as a random variable to estimate the between-cluster variation.

Secondly, multilevel modelling can estimate the cluster level effects (random effects) simultaneously with the measures of associations of community-level variables, i.e., residence, and sub-Saharan African region. Besides, previous studies conducted on factors associated with oral polio vaccination considered oral polio vaccination as a binary outcome, while oral polio vaccination status has a multinomial nature (non-vaccinated, dropout, and fully vaccinated). Therefore, treating oral polio vaccination status as binary in nature results in a loss of information and is not informative scientifically and not biologically plausible. Given the above-mentioned rationales, multilevel multinomial modelling was fitted.

The multilevel multinomial logistic regression analysis was implemented in STATA version 17 via a Generalized Structural Equation Modelling (GSEM) with the logit link and multinomial family. Four models were constructed for the multilevel multinomial logistic regression analysis. The first model was an empty model without any explanatory variables, to determine the extent of cluster variation on oral polio vaccination status categories. The second model was adjusted with individual-level variables; the third model was adjusted for community-level variables while the fourth was fitted with both individual and community-level variables simultaneously. A model with the lowest deviance was chosen.

Variables with p-value ≤ 0.2 in the bi-variable analysis for both individual and community-level factors were fitted in the multivariable model. Adjusted Relative Risk Ratio (aRRR) with a 95% Confidence Interval (CI) and p-value < 0.05 in the multivariable model were used to declare a significant association between non-vaccinated and dropout. Multi-collinearity was also checked using the generalized variance inflation factor (GVIF) which indicates that there is no multi-collinearity since all variables have VIF < 5 and tolerance greater than 0.1. Model comparison was made using deviance (-2Log-Likelihood Ratio (-2LLR)) as the models were nested and the model with the lowest deviance value was the best-fitted model.

### Ethical consideration

Ethical approval and participant consent were not necessary for this particular study since the study was a secondary data analysis based on publicly available DHS data from the MEASURE DHS program. We requested the data from the MEASURE DHS Program and permission was granted to download and use the data for this study from http://www.dhsprogram.com. There are no names of individuals or household addresses in the data files.

## Results

### Descriptive characteristics of respondents

A weighted sample of 60,846 children aged 12-23 months in SSA were included in this study. Of them, 31,527 (60.14%) of children belong to mothers aged 20-29 years and 21,845 (35.90%) of children’s mothers attained a primary level of education. About 25,388 (41.73%) of the children were in East Africa and 20,649 (33.94%) were in West Africa. About 86.59%, 81.27%, and 68.41% of children aged 12-23 months did take OPV1, OPV2, and OPV3 respectively. The prevalence of OPV dropout and fully vaccinated children in SSA were 19.38% (95% CI: 19.06%, 19.69%) and 67.77% (95% CI: 67.40, 68.14%), respectively. The dropout rate in SSA was 20.98%. More than half (61.77%) of children belonging to the poorest household and nearly three-fourths (73.87%) of children in the richest household were fully vaccinated against Polio.

**Fig 1:**
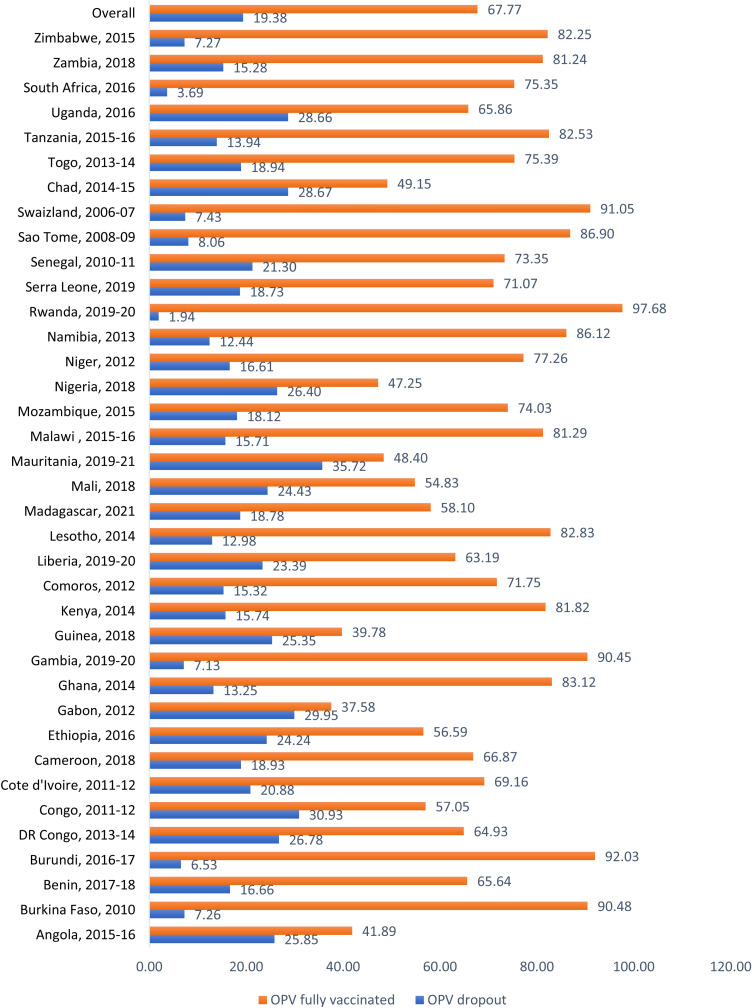
The prevalence of dropout and full vaccination of polio among children aged 12-23 months in included countries in sub-Saharan Africa.

The highest polio vaccine drop-out was observed in children born at home (22.65%), belonged to the poorest household (20.33%), born to mothers who had no formal education (20.83%), born to mothers who had no media exposure (20.82%) and resides in a rural area (20.63%) compared to their counterparts (Table 2). The prevalence of dropout and full polio vaccination varied across countries. The prevalence of dropout ranged from 1.94% in Rwanda to 35.72% in Mauritania and the prevalence of full polio vaccination ranged from 37.58% in Gabon to 97.68% in Rwanda (Fig 1).

**Table 2:** Distribution of polio vaccination status of children aged 12-23 months in included countries in sub-Saharan Africa.

**Variables**	**Vaccination status**			**Total (%)**
	**Not vaccinated** **n (%)**	**Drop out** **n (%)**	**Fully vaccinated** **n (%)**	
**Maternal age (in years)**				
15-19	830 (16.39)	1,094 (21.60)	3,142(62.01)	5,066 (8.33)
20-29	3,853 (12.22)	6,114 (19.39)	21,560 (68.38)	31,527 (60.14)
30-39	2,577 (12.75)	3,781 (18.70)	13,861 (68.55)	20,219 (33.23)
40-49	560 (13.88)	800 (19.82)	2,674 (66.30)	4,034 (6.63)
**Maternal educational status**				
No education	4.311 (20.32)	4,361 (20.55)	12,547 (59.13)	21,220 (34.87)
Primary	1,936 (8.86)	4,096 (18.75)	15,812 (72.38)	21,845 (35.90)
Secondary	1,390 (9.02)	2,927 (18.99)	11,091 (71.98)	15,408 (25.32)
Higher	183 (7.71)	405 (17.07)	1,785 (75.6)	2,372 (3.90)
**Household wealth status**				
Poorest	2,482 (17.89)	2,821 (20.33)	8,570 (61.77)	13,873 (22.80)
Poorer	1,975 (14.95)	2,613 (19.78)	8,625 (65.28)	13,213 (21.72)
Middle	1,479 (11.98)	2,375 (19.24)	8,490 (68.78)	12,343 (20.29)
Richer	1,091 (9.62)	2,143 (18.88)	8,113 (71.50)	11,348 (18.65)
Richest	793 (7.88)	1,837 (18.25)	7,438 (73.87)	10,068 (16.55)
**Place of delivery**				
Home	4,759 (24.22)	4,281 (22.65)	10,043 (53.13)	18,903 (31.07)
Health facility	3,242 (7.73)	7,508 (17.90)	31,193 (74.37)	41,943 (68.93)
**Media exposure**				
No	4,031 (18.35)	4,574 (20.82)	13,359 (60.82)	21,964 (36.10)
Yes	3,790 (9.75)	7,215 (18.56)	27,877 (71.70)	38,882 (63.90)
**Number of ANC visits (n = 57,152)**				
No ANC	2,644 (44.62)	1,361 (22.97)	1,920 (32.41)	5,925 (10.37)
1-3	1,938 (10.30)	3,624 (19.26)	13,260 (70.45)	18,822 (32.93)
≥4	2,708 (8.36)	6,011 (18.55)	23,684 (73.09)	32,404 (56.70)
**Marital status**				
Single	516 (11.63)	782 (17.62)	3,140 (70.75)	4,439 (7.29)
Married	6,819 (13.01)	10,063 (19.19)	35,545 (67.80)	52,427 (86.16)
Divorced/widowed/separated	485 (12.19)	944 (23.71)	2,551 (64.09)	3,980 (6.54)
**Husband education (n = 48,957)**				
No education	3,439 (20.64)	3,323 (19.94)	9,898 (59.41)	16,661 (34.03)
Primary	1,357 (9.23)	2,614 (17.79)	10,725 (72.98)	14,696 (30.02)
Secondary	1,423 (10.23)	2,767 (19.90)	9,715 (69.87)	13,905 (28.40)
Higher	310 (8.39)	737 (19.95)	2,648 (71.66)	3,695 (7.55)
**Sex of household head**				
Male	6,312 (13.16)	9,164 (19.11)	32,477 (67.73)	47,953 (78.81)
Female	1,509 (11.71)	2,625 (20.36)	8,959 (67.94)	12,893 (21.19)
**Parity**				
One	1,436 (11.12)	2,444 (18.91)	9,042 (69.97)	12,923 (21.24)
2-3	2,622 (11.87)	4,183 (18.93)	15,296 (69.21)	22,101 (36.32)
≥4	3,762 (14.57)	5,162 (19.99)	16,898 (65.44)	25,822 (42.44)
**Residence**				
Rural	2,201 (11.31)	4,015 (20.63)	13,242 (68.05)	19,459 (31.98)
Urban	5,620 (13.58)	7,774 (18.78)	27,999(67.64)	41,387 (68.02)
**Perceived distance to health facility (n = 55,604)**				
Not a big problem	4,424 (11.36)	7,331 (18.82)	23,655 (69.82)	38,948 (64.01)
Big problem	3,397 (15.51)	4,458 (20.36)	14,043 (64.13)	21,898 (35.99)
**Occupation**				
Working	4,431 (12.02)	6,983 (18.95)	25,430 (69.02)	36,844 (60.55)
Not working	3,390 (14.13)	4,806 (20.02)	15,806 (65.85)	24,002 (39.45)
**Sex of child**				
Male	3,916 (12.68)	6,035 (19.55)	20,923 (67.77)	30,874 (50.74)
Female	3,905 (13.03)	5,754 (19.20)	20,313 (67.77)	29,972 (49.26)
**Birth order**				
1	1,537 (11.24)	2,585 (18.90)	9,553 (69.86)	13,675 (22.47)
2-3	2,580 (11.86)	4,120 (18.94)	15,053 (69.20)	21,754 (35.75)
4-5	1,825 (13.48)	2,596 (19.18)	9,116 (67.34)	13,537 (22.25)
≥6	1,878 (15.81)	2,488 (20.94)	7,514 (63.25)	11,880 (19.53)
**Sub-Saharan African region**				
East Africa	1,747 (6.88)	4,006 (15.78)	19,635 (77.34)	25,388 (41.73)
Southern Africa	166 (8.98)	149 (8.06)	1,537 (82.97)	1,853 (3.04)
West Africa	3,462 (16.77)	4,151 (20.10)	13,035 (63.13)	20,649 (33.94)
Central Africa	2,445 (18.87)	3,482 (26.88)	7,029 (54.25)	12,956 (21.29)

### Factor associated with polio vaccination status among children aged 12-23 months

Four models were fitted (null model, model with individual-level variables, model with community variables, and model with individual and community-level variables), and the final model was the best-fitted model as it had the lowest deviance value (-2LLR) (S1 and S2 Tables). In the final multivariable multilevel multinomial logistic regression analysis; maternal age, household wealth status, maternal education status, place of delivery, media exposure, marital status, parity, residence, and sub-Saharan Africa region were significantly associated with non-vaccinated and incomplete vaccination for polio.

Children of women who attained primary, secondary, and higher education had 42%, 36%, and 25% decreased relative risks of non-vaccinated for polio compared to those born to women who had no formal education, respectively. The relative risks of non-vaccination for polio among children belonging to the poorer, middle, richer, and richest households were decreased by 9%, 18%, 26% and 30% compared to those belonging to the poorest households, respectively. The relative risks of incomplete vaccination for polio among children belonging to poorer, middle, richer and richest households were decreased by 6%, 15%, 15% and 20% compared to those belonging to the poorest households, respectively. The relative risk of being non-vaccinated among children of mothers aged 20-29, 30-39 and 40-49 years were decreased by 33%, 40% and 41% compared to women aged 15-20 years, respectively. The risks of partially vaccinated for polio among children of mothers aged 20-29, 30-39 and 40-49 years were lowered by 21%, 29% and 30% compared to mothers aged 15-20 years, respectively.

The likelihood of non-vaccinated and partially vaccinated polio children born in health facilities was decreased by 72% and 38% compared to those born to home delivery, respectively. Children born to women who had media exposure had lowered relative risks of non-vaccinated and partially vaccinated for polio by 36% and 15% compared to children born to mothers who were not media exposed, respectively. Children of currently married women have a 13% lower risk of being unvaccinated for polio compared to children of unmarried women. In contrast, children of mothers who are widowed, divorced, or separated had 34% higher risk of their children being only partially vaccinated for polio compared to mothers who have never been married (Table 3).

**Table 3:** Multilevel multinomial regression analysis of factors associated with oral polio vaccination among children aged 12-23 months in included countries in sub-Saharan Africa.

*Characteristics*	*Null model*	*Final best fitted model*	
		*Both individual and community level characteristics (aRRR with 95% CI)*	
		*Non-vaccinated*	*Incomplete*
**Maternal educational status**			
No		1	1
Primary		0.58 (0.54, 0.62)^**^	0.95 (0.90, 1.01)
Secondary		0.64 (0.59, 0.69)^**^	0.99 (0.93, 1.06)
Higher		0.75 (0.62, 0.90)^**^	1.05 (0.91, 1.20)
**Household wealth status**			
Poorest		1	1
Poorer		0.91 (0.85, 0.98)^*^	0.94 (0.88, 0.99)^*^
Middle		0.82 (0.76, 0.88)^**^	0.85 (0.80, 0.91)^**^
Richer		0.74 (0.68, 0.81)^**^	0.85 (0.79, 0.91)^**^
Richest		0.70 (0.62, 0.78)^**^	0.80 (0.73, 0.88)^**^
**Maternal age (in years)**			
15-19		1	1
20-29		0.67 (0.61, 0.78)^**^	0.79 (0.73, 0.86)^**^
30-39		0.60 (0.53, 0.68)^**^	0.71 (0.64, 0.78)^**^
40-49		0.59 (0.51, 0.69)^**^	0.70 (0.62, 0.82)^**^
**Place of delivery**			
Home		1	1
Health facility		0.28 (0.26, 0.30)^**^	0.62 (0.59, 0.65)^**^
**Media exposure**			
No		1	1
Yes		0.64 (0.61, 0.68)^**^	0.85 (0.81, 0.89)^**^
**Marital status**			
Not married		1	1
Currently married		0.87 (0.78, 0.97)^*^	1.02 (0.93, 1.12)
Divorced/widowed/separated		1.02 (0.88, 1.18)	1.34 (1.19, 1.50)^**^
P**arity**			
One		1	1
2-3		1.11 (1.02, 1.21)^*^	1.07 (1.00, 1.14)^*^
≥4		1.09 (0.99, 1.20)	1.16 (1.07, 1.25)^**^
**Residence**			
Urban		1	1
Rural		0.73 (0.68, 0.78)^**^	0.89 (0.84, 0.94)^**^
**sub-Saharan Africa region**			
East Africa		1	1
Southern Africa		1.47 (1.22, 1.77)^**^	0.61 (0.52, 0.73)^**^
Central Africa		3.78 (3.52, 4.05)^**^	1.67 (1.58, 1.76)^**^
West Africa		2.56 (2.39, 2.74)^**^	2.59 (2.45, 2.73)^**^

** *aRRR: adjusted Relative Risk Ratio, CI: Confidence Interval,*

* *p-value < 0.05,*

** *p-value < 0.01.*

Children born to mothers who had 2-3 births had 11% (aRRR = 1.11, 95% CI: 1.02, 1.21) higher relative risk of not having polio vaccination compared to primiparous women while children born to mothers who had 2-3 births, and four and above were 7% (aRRR = 1.07, 95% CI: 1.00, 1.14) and 16% (aRRR = 1.16, 95% CI: 1.07, 1.25) higher relative risk of partial vaccination for polio than primiparous women, respectively. The relative risk of non-vaccinated and partially vaccinated for polio among rural children was decreased by 27% (aRRR = 0.73, 95% CI: 0.68, 0.78) and 11% (aRRR = 0.89, 95% CI: 0.84, 0.94) compared to urban children.

Children in Southern Africa, Central Africa, and West Africa had 47% (aRRR = 1.47, 95% CI:1.22, 1.77), 3.78 (aRRR = 3.78, 95% CI: 3.52, 4.05) and 2.56 times (aRRR = 2.39, 95% CI: 2.39, 2.74) higher relative risk of non-vaccinated for polio compared to children in East Africa, respectively. The relative risk of non-vaccination among children in Southern Africa decreased by 39% (aRRR = 0.61, 95% CI: 0.52, 0.73) compared to children in East Africa while children in Central Africa and West Africa had 67% (aRRR = 1.67, 95% CI: 1.58, 1.76) and 2.59 times (aRRR = 2.59, 95% CI: 2.45, 2.73) compared to children in East Africa, respectively (Table 3).

## Discussion

This study investigated the prevalence of polio vaccination and factors associated with drop-out among children aged 12–23 months in sub-Saharan Africa. This new knowledge is important to help guide vaccination-related policies, interventions and actions as the global community pushes to eliminate polio globally. Our result showed a full vaccination prevalence of 67.77% and a drop-out prevalence of 19.38%. The result of full vaccination indicates that only about 3 in 5 children aged 12–23 months have a full dose of OPV. This vaccination prevalence is worrisome as it is likely to defeat the gains that have been recorded in eradicating polio from sub-Saharan Africa. With only about 3 in 5 children being fully vaccinated, the chances of the resurgence of polio may be high. This resurgence may also be reinforced by a relatively high drop-out rate which our results peg at 1 in 5 children.

Interestingly, our results reveal notable between-country disparity in the proportion of children who are fully vaccinated and those who drop out. For instance, while Rwanda recorded 97.68% full vaccination and 1.94% drop-out in their 2019–2020 data, Mauritania reported only 48.4% full vaccination and 35.72% drop-out in their 2019–2021 data. Similarly, there was also disparity in non-vaccination by regions within the sub-Saharan African region with Southern Africa having the least chance of non-vaccination followed by West Africa and Central Africa in that order. This disparity by region may be attributable to both economic and socio-political instability reasons. For instance, Central Africa is home to some African countries that are known to be very volatile, for instance, Chad. It is not unlikely that this volatility would impact childhood vaccination, including polio vaccination. Our result shows that more than one in four children in Chad drop out or are not vaccinated at all against polio – one of the highest proportions in the countries included in our analysis. High proportion of drop-out or non-vaccination is also recorded for other Central African countries included in our analysis such as Angola, Gabon, DR Congo and Congo. Achieving and/or sustaining polio eradication targets both in sub-Saharan Africa and globally requires equitable and “one health” strategies as well as commitments from regional and national governments to own, implement and evaluate these strategies for desired results. There is evidence of polio re-emergence in high-income countries such as the United States and United Kingdom [8–10]. In sub-Saharan Africa, countries like Rwanda, Burkina Faso, Burundi, Gambia and Swaziland with at least 90% full vaccination could serve as role models, having attained vaccination at least 9 in 10 children.

Our results showed that maternal age, household wealth status, maternal education status, place of delivery, media exposure, marital status, parity, residence, and sub-Saharan Africa region were significantly associated with non and incomplete vaccination for polio. Our result suggests that children born to adolescents and young women aged 15–19 years were more likely to be partially vaccinated or unvaccinated. This implies that children born to adolescents may be more likely to experience poorer health outcomes such as non or vaccination drop-out. This could be attributable to the likelihood of these adolescent mothers not being in marriages and the social exclusion, stigma and embarrassment that they may be experiencing [41,42]. The socio-economic and socio-cultural burden of teenage and/or adolescent pregnancies could significantly impact the health literacy and social capital of these adolescent mothers thereby leading to poorer health outcomes for their children including under-vaccination [42]. Even when adolescent motherhood occurs within the context of marriage, there is still the likelihood of structural barriers hampering the extent to which these adolescent mothers can provide necessary care and vaccination for their children. Thus, there is a need to put necessary measures in place in sub-Saharan Africa to drastically reduce, and if possible, eradicate teenage marriages and unplanned pregnancies among adolescent girls.

Also, we found that children born to mothers without formal education were more likely to be unvaccinated against polio or to have dropped in the vaccination chain. Formal education is important in improving health literacy [43] and knowledge which is critical in health-seeking behaviour including vaccination against childhood diseases [44,45]. Aside from health literacy, formal education enhances social capital that may be closely tied to the affordability and accessibility of health services and childhood vaccination [46]. Little wonder we also found that non-vaccinated and partially vaccinated children against polio were from the poorest households. Mother’s education is also closely tied to media access and exposure as women who are formerly educated are more likely to have higher media exposure. This exposure has the tendency of increasing health awareness and literacy which invariably increases the likelihood of engaging with available health services including childhood vaccination [47–49]. This tendency is well reflected in our results as non-vaccinated or partially vaccinated children were more likely to be from homes where women have no media exposure. The implication of these results puts women’s education at the forefront of interventions and policies to enhance children’s health outcomes and wellbeing.

In line with evidence [44–47,49,50], our findings show that illiteracy and low socio-economic status resulting in low women empowerment remain significant factors in low childhood vaccination uptake. Functional education that also incorporates sexual and reproductive health could be an effective pathway to better women’s empowerment. Functional education is protective against unplanned pregnancy in adolescence and enhances economic and social productivity in adulthood. When adolescent girls complete their educational cycle, they are less likely to experience teenage pregnancy as schooling years enable them to enter marriage at a more appropriate age [50]. This is especially the case when active sexual and reproductive health education is part of the curriculum. Therefore, functional education including sexual and reproductive health education, could be a silver bullet that could improve the health and wellbeing of children regarding vaccination against childhood diseases.

### Strengths and limitations

This study provides empirical evidence from 37 countries using robust analytical techniques, specifically multilevel multinomial regression. This model is particularly well-suited for analysing complex, hierarchical data with categorical outcomes, providing robust and nuanced insights while accounting for the nested structure of the data [1,2]. We employed a multilevel multinomial logistic regression model, a robust analytic technique for analysing categorical outcomes with multiple unordered categories in hierarchical data structures. This model examined polio vaccination status (non-vaccinated, dropout, and fully vaccinated) by considering the nested structure of the data, where observations are clustered within higher-level units. It yields more accurate estimates of effects and standard errors compared to standard multinomial logistic regression, which may not adequately handle such multi-categorical outcomes with hierarchical characteristics. Additionally, these models incorporate random effects to account for unobserved heterogeneity at higher levels, capturing variations in outcomes across clusters that are not explained by the measured predictors. The DHS employs a representative sampling approach, making the findings, conclusions, and recommendations generalizable to children aged 12-23 months in the studied countries. However, as this is a cross-sectional study, causality cannot be inferred. Additionally, since the data on children’s immunization sources were based on verbal reports from mothers, there is a possibility of underreporting or overreporting, and a possibility of recall and social desirability bias. These could overestimate or underestimate the proportion of polio vaccination status, and which in turn influence the effect size [3].

## Conclusion

Several factors contribute to children missing polio vaccinations or receiving incomplete doses. These include a mother’s level of education, the family’s economic status, the mother’s age, the place where the child was delivered, exposure to media, marital status, the number of children a mother has, and whether the family lives in an urban or rural area. These findings highlight the need for focused and practical interventions to address these challenges and improve vaccination rates across sub-Saharan Africa.

Governments, particularly in Western and Central Africa, should consider several approaches. Educating mothers through community-based programs can help them understand the importance of polio vaccination and how it protects their children. Making health services more accessible by improving infrastructure and transportation in rural areas is also essential. Local media campaigns can be a powerful tool to inform the public about vaccination schedules and benefits, raising awareness and encouraging participation.

Healthcare workers play a crucial role in this effort and supporting them with better training and incentives can strengthen their ability to engage with communities and promote immunization. Addressing financial barriers is equally important, and programs that help low-income families cover the costs of transportation or clinic visits can make vaccinations more accessible.

By implementing these strategies, governments can improve polio vaccination coverage, reduce the risk of outbreaks, and ensure better health outcomes for children in the region.

## Supporting information

S1 Table Model comparison parameters (DOCX)

S2 Table Multilevel multinomial regression analysis of factors associated with oral polio vaccination among children aged 12-23 months in included countries in sub-Saharan Africa (DOCX)
