# Supplementary material for: Unveiling the Drivers of Polio Vaccine Uptake: Insights from a Multi-Country Study of 37 Nations in Sub-Saharan Africa
Source: PLoS One. 2025 Mar 19;20(3):e0316884. doi: 10.1371/journal.pone.0316884 (PMC11922275; doi:10.1371/journal.pone.0316884)
Supplement: S1 Table — (DOCX) [file pone.0316884.s001.docx]

**Table S1. Model comparison parameters**

| **Models** | **Deviance (-2LLR)** | **AIC** | **BIC** |
| --- | --- | --- | --- |
| Null model | 104,418.66 | 104,425.9 | 104,452.9 |
| Model 1 | 99,115.8 | 99,185.8 | 99,501.78 |
| Model 2 | 100,663.84 | 100,685.8 | 100,785.1 |
| Model 3 | 96,521.54 | 96,607.55 | 96,995.76 |

^*^AIC: Akaike Information Criteria, BIC: Bayesian Information Criteria, LLR: Log-likelihood Ratio
