# Supplementary material for: Unveiling the Drivers of Polio Vaccine Uptake: Insights from a Multi-Country Study of 37 Nations in Sub-Saharan Africa
Source: PLoS One. 2025 Mar 19;20(3):e0316884. doi: 10.1371/journal.pone.0316884 (PMC11922275; doi:10.1371/journal.pone.0316884)
Supplement: S2 Table — (DOCX) [file pone.0316884.s002.docx]

**Table S2. Multilevel multinomial regression analysis of factors associated with oral polio vaccination among children aged 12-23 months in included countries in sub-Saharan Africa**

| **Characteristics** | **Null model** | **Model I** | | **Model II** | |
| --- | --- | --- | --- | --- | --- |
|  |  | Individual level variables (aRRR with 95% CI) | | Community level variables (aRRR with 95% CI) | |
|  |  | Non-vaccinated | Incomplete | Non-vaccinated | Incomplete |
| **Maternal educational status** | | | | | |
| No |  | 1 | 1 |  |  |
| Primary |  | 0.45 (0.43, 0.48) | 0.82 (0.78, 0.87) |  |  |
| Secondary |  | 0.61 (0.56, 0.66) | 0.96 (0.91, 1.03) |  |  |
| Higher |  | 0.66 (0.55, 0.80) | 0.95 (0.83, 1.09) |  |  |
| **Household wealth status** | | | | | |
| Poorest |  | 1 | 1 |  |  |
| Poorer |  | 1.02 (0.95, 1.09) | 0.99 (0.93, 1.05) |  |  |
| Middle |  | 0.96 (0.89, 1.04) | 0.94 (0.88, 0.99) |  |  |
| Richer |  | 0.91 (0.84, 0.99) | 0.94 (0.88, 1.01) |  |  |
| Richest |  | 0.91 (0.82, 1.01) | 0.91 (0.84, 0.98) |  |  |
| **Maternal age (in years)** | | | | | |
| 15-19 |  | 1 | 1 |  |  |
| 20-29 |  | 0.62(0.56, 0.68) | 0.73 (0.67, 0.79) |  |  |
| 30-39 |  | 0.53 (0.48, 0.60) | 0.63 (0.57, 0.70) |  |  |
| 40-49 |  | 0.52 (0.45, 0.60) | 0.62 (0.55, 0.70) |  |  |
| **Place of delivery** | | | | | |
| Home |  | 1 | 1 |  |  |
| Health facility |  | 0.28 (0.26, 0.29) | 0.61(0.58, 0.64) |  |  |
| **Media exposure** | | | | | |
| No |  | 1 | 1 |  |  |
| Yes |  | 0.66 (0.62, 0.69) | 0.84 (0.80, 0.88) |  |  |
| **Marital status** | | | | | |
| Not married |  | 1 | 1 |  |  |
| Currently married |  | 0.82 (0.74, 1.02) | 1.05 (0.96, 1.14) |  |  |
| Divorced/widowed/separated |  | 0.88 (0.77, 1.02) | 1.33 (1.19, 1.49) |  |  |
| P**arity** | | | | | |
| One |  | 1 | 1 |  |  |
| 2-3 |  | 1.17 (1.08, 1.28) | 1.12 (1.05, 1.20) |  |  |
| ≥4 |  | 1.20 (1.09, 1.32) | 1.28 (1.18, 1.38) |  |  |
| **Residence** | | | | | |
| Urban |  |  |  | 1 | 1 |
| Rural |  |  |  | 1.53 (1.45, 1.62) | 1.15 (1.10, 1.20) |
| **sub-Saharan Africa region** | | | | | |
| East Africa |  |  |  | 1 | 1 |
| Southern Africa |  |  |  | 1.13 (0.95, 1.36) | 0.57 (0.49, 0.67) |
| Central Africa |  |  |  | 4.98 (4.65, 5.33) | 1.74 (1.65, 1.83) |
| West Africa |  |  |  | 3.35 (3.15, 3.57) | 2.85 (2.70, 3.01) |

*** aRRR: adjusted Relative Risk Ratio, CI: Confidence Interval,*
